# Assessing Households Preparedness for Earthquakes: An Exploratory Study in the Development of a Valid and Reliable Persian-version Tool

**DOI:** 10.1371/currents.dis.ccc8697279713e66887b928b839d0920

**Published:** 2016-02-25

**Authors:** Ali Ardalan, Sanaz Sohrabizadeh

**Affiliations:** School of Public Health, Disaster and Emergency Health Academy, National Institute of Health Research, Tehran University of Medical Sciences, Tehran, Iran; Harvard Humanitarian Initiative, Harvard University, Cambridge, USA; Department of Health in Disasters and Emergencies, School of Health, Safety and Environment, Shahid Beheshti University of Medical Sciences, Tehran, Iran

## Abstract

Introduction: Iran is placed among countries suffering from the highest number of earthquake casualties. Household preparedness, as one component of risk reduction efforts, is often supported in quake-prone areas. In Iran, lack of a valid and reliable household preparedness tool was reported by previous disaster studies. This study is aimed to fill this gap by developing a valid and reliable tool for assessing household preparedness in the event of an earthquake.

Methods: This survey was conducted through three phases including literature review and focus group discussions with the participation of eight key informants, validity measurements and reliability measurements. Field investigation was completed with the participation of 450 households within three provinces of Iran. Content validity, construct validity, the use of factor analysis; internal consistency using Cronbach's alpha coefficient, and test-retest reliability were carried out to develop the tool.

Results: Based on the CVIs, ranging from 0.80 to 0.100, and exploratory factor analysis with factor loading of more than 0.5, all items were valid. The amount of Cronbach's alpha (0.7) and test-retest examination by Spearman correlations indicated that the scale was also reliable. The final instrument consisted of six categories and 18 questions including actions at the time of earthquakes, nonstructural safety, structural safety, hazard map, communications, drill, and safety skills.

Conclusion: Using a Persian-version tool that is adjusted to the socio-cultural determinants and native language may result in more trustful information on earthquake preparedness. It is suggested that disaster managers and researchers apply this tool in their future household preparedness projects. Further research is needed to make effective policies and plans for transforming preparedness knowledge into behavior.

## Introduction

Earthquakes strike quickly, without any warning and are uncontrollable disasters, making the main cause of injuries, deaths, and the destruction of physical infrastructures 1. Worldwide evidence reveals that there are upward trends in the number of individuals who are affected by earthquakes 2.Based on the World Disaster Report (2013), Iran has been placed among countries suffering from the highest number of casualties at the time of an earthquake 3. For example, the 1990 Rudbar-Manjil earthquake claimed 35000 fatalities and the Bam quake of 2013 killed roughly 41000 inhabitants – standing as the most deadly natural disasters in Iranian history 4. Accordingly, the sudden and persistent nature of quakes reflects the need for proper preparation 5.

Preparedness is defined as the activities and measures taken in advance to ensure effective response to the destructive impacts of disasters. Earthquake preparedness is considered a significant way to reduce the risk of quakes 6 ^,^ 7. The preparedness actions may include collecting survival items, planning what households do at the time of earthquakes, and mitigation activities 8 ^,^ 9 ^,^ 10. In terms of preparedness, strategies may be administered at either the community or individual level 11.

At the individual level, household preparedness is one element of risk reduction efforts that is often supported in quake-prone areas 5. The preventive measures increase coping capacity as well as community resilience for recovering from the ramifications of quakes 12. A prepared household can take care of itself for the first seventy-two hours after an earthquake. Households, as the first responders, usually focus on restoring lifelines and the most acute rescue needs prior to the arrival of outside assistance 13.

To estimate the level of household preparedness, a clear identification of those prepared and those unprepared may be necessary 14. Consequently, using a questionnaire is one of the most important and acceptable ways for gathering data 15. Reliability and validity criteria are the key quality indicators for measuring tools 16. A valid and reliable instrument can guarantee relevant and accurate assessments of household preparedness in the event of an earthquake.

A number of authors have developed valid and reliable tools for measuring preparedness. Some investigations used brief measures 17 ^,^ 18 and others applied longer scales to assess earthquake preparation 19 ^,^ 20. For instance, the new index of public preparedness in the event of an earthquake was developed to estimate preparation levels of Japanese and American communities 21. In New Zealand, the English version of earthquake readiness scale (ERS) was established to evaluate levels of public preparation, including emergency kit and standard building constructions 14. Additionally, psychological and demographic antecedents of survival and mitigation activities were investigated 22. Similarly, survival, planning, and hazard mitigation factors were identified through the development of an earthquake preparedness scale 23. As any instrument should be developed in accordance with socio-cultural determinants and language diversities 24, a valid and reliable Persian tool was developed to fill the following gaps:

First, significant attention should be paid to assess household preparation against upcoming earthquakes, as many Iranian households have been increasingly victimized by quakes. Hence, developing an evaluation instrument for measuring household preparation against quakes can be the start point of any mitigation initiative. Second, all relevant tools have been developed in English. In Iran, there have been no valid and reliable Persian tools that can be applied by disaster scholars, researchers and practitioners. This gap was highlighted by earthquake mitigation studies, which were conducted in the absence of a valid and reliable Persian tool 2 ^,^ 25 .

Developing a context-based instrument has been recommended by disaster researchers in Iran to resolve this barrier. Third, disaster scholars from various nationalities (Arabic, Korean and etc) have developed their own native-language tool in line with the necessary contexts. Emergency kit items and household communication may be context-based elements. For instance, in Iran and nations with similar religious and cultural backgrounds, a separate and private place should be provided for women who are involved in disaster prevention activities. This is largely because gender-based relations and cultural norms can limit risk communication within households and should be considered in the development of questionnaires and guidelines. Finally, a simple and comprehensive Persian tool can be applied by a number of Persian speaking nations that have not yet developed any valid and reliable preparedness instruments. For example, our Persian tool may be used in Tajikistan, which has similar language and context.

Considering these gaps, the present survey is aimed to explore a valid and reliable Persian tool to evaluate earthquake preparedness at the household level. Relevant and accurate evaluation provided by such a valid and reliable instrument can result in appropriate disaster planning and policy-making. Reducing the rates of loss of life and improving community survival are the final goals of using a valid and reliable preparedness tool.

## Methods

**Setting**

Iran (the Islamic Republic) is largely affected by natural and human-made disasters. The country is situated in the Alpe–Himalayan earthquake belt, characterized by high seismic activity 26.

This study was performed in three provinces, namely in Kurdistan, Zanjan, and South Khorasan. Since these provinces are demonstrations of three dominant cultural contexts of Iran, our tool can be more applicable for the majority of citizens. Furthermore, all selected provinces are located on seismic belts with active fault. Their histories showed considerable destructions, due to previous earthquakes. For example, the earthquake of Rudbar-Manjil in the Zanjan province killed 35,000 of citizens and damaged more than 200,000 houses 27. A number of seismic surveys reported that these three provinces are quake-prone regions 28.

**Questionnaire Development**

The questionnaire was developed through three phases:

1) Literature review and focus group discussions

2) Measurement of the tool validity

3) Measurement of the instrument reliability

**Phase One: Literature Review and Focus Group Discussions**

A literature review was carried out to prepare the basic framework of the tool. Several databases including PubMed, Google Scholar, Scopus, FEMA archive, Red Crescent and UNISDR were searched. The following search strategy and keywords were used:

(Earthquake* OR quake) AND (prepare* OR readiness OR mitigation) AND (questionnaire* or tool* or scale*) AND (household* OR population* OR citizen*).

Included documents were considered for preparing the primary content of the tool. Translation validity was achieved by translating the relevant items into Persian (forward translation) and then back to English (backward translation), conducted by an applied linguist.

Two focus group discussions using the purposive sampling were carried out to modify the primary framework in regards to the socio-cultural context and language diversity. A total of eight key informants participated in the focus group discussions. Participants from different disciplines were invited, including disaster epidemiology (two experts), disaster health management (three specialists), disaster medicine (one expert), and earthquake studies (two researchers). In addition, the specialists had useful disaster experiences, as they had previously served as service providers and disaster investigators. Each focus group included a structured open-ended discussion for basic items and room for suggestions for other relevant items. Since the key informants came from dominant cultural contexts in Iran (Kurd, Azeri and Fars), they considered socio-cultural factors in their discussions. During the first focus group, 15 items were derived from the literature review and were discussed in line with the socio-cultural factors. Then, 15 items were added to the list, according to the participants’ suggestions. During the second focus group, all 30 items were discussed and 20 items were selected to be form as a question. At the end of this stage, all 20 items were merged into 18 questions.

**Participants**

During the following phases, a field survey was completed with the participation of 450 households living in Kurdistan, Zanjan and South-Khorasan. Since including all cities and villages of the three provinces was not possible granted our funds and facilities, one city and one village were selected randomly on the map. Random selection was applied in order to give all quake-prone regions an equal chance to be chosen for surveying, as well as generalization of the findings.

In the next stage, 75 urban and 75 rural households were selected in each province. The samples were chosen by systematic random sampling of the registration list of Family Physicians in health centers within each province. The respondents of the questionnaires were mothers/housewives, because they often play central roles in the family and have more information about household affairs. If she had not been at home, other family members would have been asked to answer the questions.

**Phase Two: Measuring Validity**

Content Validity: A panel of experts was used to establish content validity of the final items, which was carried out through a self-completion questionnaire. In this phase, 10 experts from various disciplines of disaster management participated in the panel. Discussions were led by two moderators who used a checklist of questions with four-scale scoring to achieve the Content Validity Index (CVI) (ranging from not related (=1) to completely related (=4)). Based on the CVI index, a rating of three or four indicates that content is valid and consistent with the conceptual framework 29.

Construct Validity: Construct validity can be determined by factor analysis 30. Exploratory factor analysis (EFA), as a statistical method, was applied to develop the instrument by clustering items into common factors. Principal component analysis (PCA) and Varimax methods were used for undertaking EFA. To ensure an appropriate sample size for running the factor analysis, the Kaiser-Meyer-Olkin (KMO) sampling adequacy on the scale was computed. The accepting values of ≥ 0.5 were recommended.

**Phase Three: Measuring Reliability**

Internal Consistency Reliability: Internal consistency examines the inter-item correlations within an instrument 30. Cronbach’s alpha was computed for the whole scale to examine its internal consistency.

Test-retest Reliability: Test-retest reliability was undertaken by administrating the questionnaire in 25 households, randomly selecting from the first 75 household samples. Two months was considered as a suitable interval time between the two tests. Spearman correlation was carried out to determine whether there is any significant relationship between the responses at each time point.

All the questioners were trained and informed about the methods of gathering data in the fields. The training subjects included sampling methods, motivating samples for participation, ethical considerations, communication and etc. Additionally a guideline was prepared for explaining study objectives, sample sizes, sampling methods, household numbers, questionnaire codes, coding response items, and communication.

**Ethical consideration**

The study was approved by the ethical committee of Tehran University of Medical Sciences, Tehran, Iran. All participants were informed about the confidentiality of their names in related reports and final publications. Based on the consent form, the participants were provided the possibility of leaving or declining their participation.

## Results

Based on the CVI results, all valid items ranged from 0.80 to 0.100. The KMO sampling adequacy on the tool was 0.87 which indicated the sample size of 450 had been adequate for performing factor analysis.

Regarding construct validity, total variance of the factors was 71% on the first run of PCA, in which at least 50% of the variance could be explained by common factors 31. Among two to six factor solutions, a six factor solution with the Varimax rotation was assumed to be the most appropriate to the scale. To undertake the most suitable interpretation, the loading values were carefully examined using the guideline for practical significance. This guideline shows that a factor loading of 0.5 means the factor is significant 32. As a result, all items with a loading of ≥ 0.5 were accepted. Factor loadings of the final PCA and their factorial weights are shown in Table 1.

**Figure 1:**
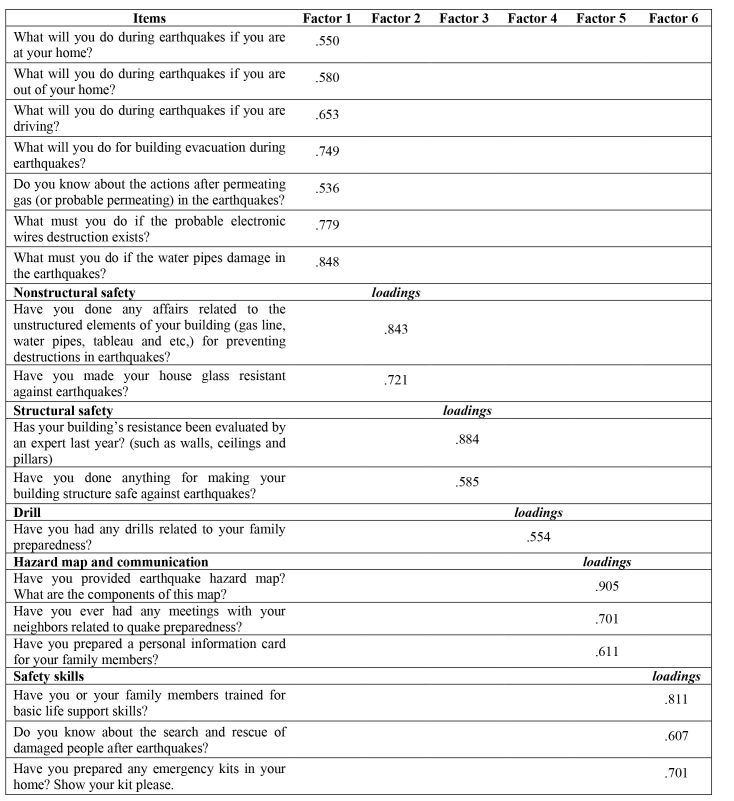
**Table 1: Final seven factor solution of the scale according to the Principal Component Analysis with Varimax rotation**

Internal consistency reliability of the questionnaire was examined by Cronbach’s alpha. The computed alpha was 0.7, which revealed that there is an acceptable correlation between the items and whole questionnaire. Spearman Non-parametric statistical test showed significant differences between data extracted from test-retest stage (Table 2). That is, the tool benefited from external reliability as well.

**Figure 2:**
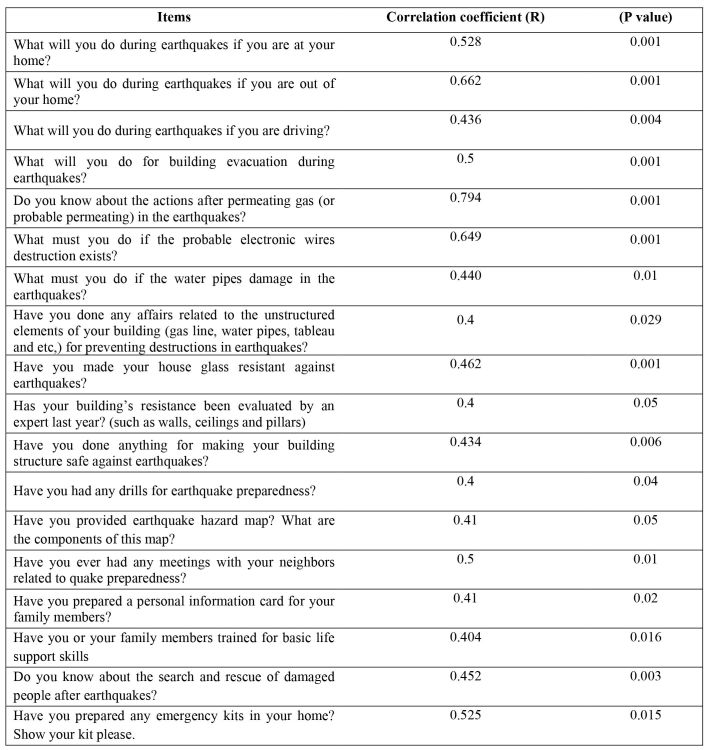
**Table 2: Test-Retest results of the Scale using Pearson non-parametric test.**

**The Final Instrument**

The first part of the questionnaire consists of three sections: questioner’s information, respondent demographic/economic information, and household experiences on earthquakes. The main six categories and 18 questions have been described below:

Factor one: “Actions at the time of earthquakes” was accounted for 34.3% of the total variance. This factor contains seven questions and reflects information on how much households know about the necessary actions at the time of earthquakes. The highest loading item was: “What must you do if the water pipes become damaged in the event of an earthquake?”(with a factor loading of 0.84).

Factor two: “Nonstructural safety” accounted for 11.97% of the total variance. It explores the household actions for nonstructural elements of buildings, such as heavy materials. The item, “What have you done about the nonstructural elements of your buildings which can damage you?” had the highest loading of 0.843.

Factor three: “Structural safety” was accounted for 7.18% of the variance, which highlights the necessity of structural factors for preventing earthquakes destructions. “Assessing the structural elements of building by the experts” had the highest loading of 0.815.

Factor four: “Drill” was accounted for 6.22% of the total variance. This factor contains one item and includes preparedness drills regarding evacuation, hazard maps, and so on.

Factor five: “Hazard map and communication” accounted for 5.83% of the variance reflecting personal information card, hazard map and household communication with their neighbors. The hazard map portion had the highest loading of 0.905.

Factor six: “Safety skills” accounted for 5.5% of variance. This includes household knowledge on basic life support skills (with the highest factor loading of 0.811), rescue skills, and preparing an emergency kit.

There were predefined responses to the questions ranging from two to nine items. To check the preparedness behavior of households, the participants were asked to show the emergency kit, hazard map, and information card if it was already prepared. The coding method was completely explained in the guideline.

## Discussion

Developing a valid and reliable Persian tool has been the first scientific attempt to estimate the extent of household preparedness in the event of an earthquake. The validity and reliability features of the tool indicate that it is accurate enough to evaluate different aspects of household preparedness in the quake-prone regions.

Lack of a valid and reliable preparedness tool for Iran was the main motivator in developing the present questionnaire. In Iran, a number of previous household preparedness investigations were not conducted using a valid and reliable tool 2 ^,^ 25. Since we considered the concerns of major cultural groups of Iran in developing the tool, the questionnaire is hoped to be used by the majority of disaster scholars and practitioners to successfully assist improve the field of disaster management studies. In addition, Farsi-speaking countries such as Tajikistan may use this tool for assessing household preparedness against earthquakes.

Some studies successfully reported amounts of household preparedness in the event of a natural disaster; however, lack of a valid and reliable assessment tool was obvious in their reports 17 ^,^ 18 ^,^ 19 ^,^ 20. For example, the preparedness survey of Emergency Management Queensland was conducted by applying items such as emergency kit preparation, emergency plan, and home maintenance 19. Although these studies yielded significant insights into the general population’s preparation in natural disasters, just a few reports included valid and reliable scales for measuring household preparedness. Consequently, possible measurement errors concerning preparedness tools makes interpretation of the findings limited. In the following paragraphs, a number of important English-version preparedness tools have been discussed.

In accordance with our findings, the earthquake readiness scale (ERS) has been developed by the same methodology used in the present study. Based on the ERS report, survival and mitigation items of the tool were identified and developed through a community-based survey 14. It seems that validation criteria were highlighted more than reliability measures of the ERS. In addition, a number of preparedness factors, such as risk communication and drill and hazard map, have been more centralized in the development of our preparedness tool.

The other English version earthquake preparedness scale consisted of three factors including survival, planning, and hazard mitigation. The development process of this tool was completed through a community-based survey in two affected regions 23. Hazard mitigation is equal to structural and nonstructural safety categories of our tool, and the survival factor is similar to safety skill factors. Using a Persian version tool adjusted to the socio-cultural determinants may result in more trustful information on household preparedness. For example, since Iran is a religious country, the Quran is usually considered in preparing the emergency kit. Furthermore, regarding risk communication, the necessity of providing a separate and private space for women is obvious. We have tried to consider such cultural factors in the questionnaire and its guidelines.

A number of limitations were identified during the study process. One limitation was related to physical distance between the investigators and the questioners. As the authors were citizens of Tehran and the questioners lived in Zanjan, Kurdestan and South Khorasan, training the questioners for data gathering was a time-consuming process. Coordination for data gathering was the second limitation, as the three provinces had to start and finish data collections at the same time. To control this limitation, we asked the senior health officials in each province to supervise the data gathering process. Finally, at the final stage of data analysis, we could not run confirmatory factor analysis (CFA), as a consequence of inadequate sample size for CFA. Limited budget and facilities restricted us from selecting more samples. However, this cannot prohibit stakeholders from using the tool, because the results support the validity and reliability of the questionnaire.

## Conclusion

The negative consequences of earthquakes can be reduced by preparing households appropriately in the event of quakes. Considering the high capacity of public health networks distributed around the country, health community workers can successfully apply the tool for estimating household preparedness. Primary health centers are active community-based health agencies in Iran, so it is strongly recommended to use their full participation to accomplish any disaster preparedness and mitigation projects.

Achieving research integrity can be assumed as one of the main advantages of the present study. Hence, it is suggested that disaster managers and researchers apply this tool in their future household preparedness projects. On the other hand, performing different pilot studies and using a larger sample size are suggested steps in future disaster research projects.

Based on our investigation, turning the knowledge of preparedness into practice may be one of the most important challenges in earthquake preparedness. The present tool can evaluate both knowledge and practice, regarding certain aspects of household preparedness. For example, although participants had confirmed that they prepared the emergency kit, the questioners asked them to bring and present the kit. In some cases, the participants had inaccurately reported their preparedness, but this follow-up system allowed for understanding these inconsistencies. Accordingly, further research is necessary to identify effective plans to improve preparedness behavior. Finally, lack of a disaster prevention culture in Iran can be a challenge for any preparedness initiative, as prevention methods are often not taken seriously. Therefore, a significant question we must answer in the future is, "how can disaster preparedness be culturally established among Persian households?"

## Competing Interests

The authors have declared that no competing interests exist.

## Corresponding Author

 Sanaz Sohrabizadeh is the Corresponding Author for this paper and can be reached at ssohrabizadeh@gmail.com.
